# Selective nerve root block combined with posterior percutaneous cervical endoscopic discectomy for cervical spondylotic radiculopathy with double nerve root variation: A case report

**DOI:** 10.1097/MD.0000000000019464

**Published:** 2020-03-20

**Authors:** Tong Yu, Jun Zhang, Jiu-Ping Wu, Hai-Chi Yu, Hai-Qing Tian, Su-Li Luo, Qin-Yi Liu

**Affiliations:** Department of Orthopedics, The Second Hospital of Jilin University, Changchun, Jilin Province, China.

**Keywords:** cervical disc herniation, endoscopic, nerve root blockage, percutaneous

## Abstract

**Rationale:**

The aim of this report is to present the technique of selective nerve root blockage combined with posterior percutaneous cervical endoscopic discectomy (PPECD) for cervical spondylotic radiculopathy (CSR).

**Patient concerns:**

A 49-year-old female has pain in the skin area of the left scapular, pain in left elbow and limitation of left upper limb movement for 1.5 years.

**Diagnosis:**

She was diagnosed with CSR and C6-7 double nerve root variation.

**Interventions:**

We used selective nerve root block to determine the lesion segment and applied PPECD to relieve pressure on the patient's nerve roots.

**Outcomes:**

The pain symptoms disappeared after the patient was treated with C6-7 nerve root block. Endoscopic displayed C6-7 double nerve root variation on the left side of the spinal cord intraoperative. The neurological function was intact postoperatively and no recurrence of cervical disc herniation during the 5 months’ follow-up period. The hospitalization time was 5 days, the operation time was 68.2 minutes and the bleeding volume was 52.6 ml. There was no change in cervical curvature and cervical disc height postoperatively. Japanese Orthopaedic Association score, SF-36 score and Visual Analogue Scale score improved significantly postoperatively.

**Lessons:**

The application of selective nerve root blockage combined with PPECD for CSR could achieve satisfactory effect of position and decompression of the injured nerve root. Besides, we recommend that surgery be performed under general anesthesia to minimize patients’ emotional stress and discomfort.

## 1 Introduction

Cervical spondylotic radiculopathy (CSR) is usually presented with a pain, numbness, or tingling in the upper extremity to electrical type pain or even weakness, which is caused by compression of a cervical nerve root.^[1–4]^ The responsible segment is usually determined by combining medical history, physical examination, signs, and imaging examination. However, the results of clinical examinations are often inconsistent with those of imaging examinations. It is incorrect to decompress blindly according to the pressure shown on imaging, which may lead to no improvement of symptoms after operation. Therefore, how to determine the responsible intervertebral space becomes a key issue for the treatment of atypical cervical disc herniation (CDH). Park et al reported that selective nerve root block has been used for conservative management of cervical radicular pain and obtained satisfied outcome.^[5]^

Conservative treatment is initially indicated for patients that have degenerative cervical radiculopathy, and surgical treatment is recommended when conservative treatment is ineffective.^[6]^ Endoscopic decompression obviates the need for muscle dissection and disruption of the posterior tension band that can prevent postlaminectomy kyphosis.^[7]^ It does not require sacrifice of a cervical motion segment. Lower incidence of complications, and rapid recovery from unrestricted activity minimal blood loss, less surgical time, short X-ray time, and hospital stay^[8,9]^ are the advantages compared with open techniques. To our best knowledge, posterior percutaneous endoscopic cervical discectomy (PPECD) for CSR with double nerve root variation has rarely been reported. We report our initial experience of selective nerve root blockage (SNRB) combined with PPECD for CSR.

## 2 Ethics

This report was approved by the ethics committee of the Second Hospital of Jilin University, Changchun, China. The patient provided written informed consent for this report, and his information has been anonymized.

## 3 Case report

### 3.1 Patient characteristics

This otherwise healthy female patient is 49 years old. She has pain in the skin area of the left scapular, pain in left elbow and limitation of left upper limb movement for 1.5 years; besides, the symptoms of discomfort have been worse for 2 weeks.

Examination revealed the significant skin pain in the left scapular region and elbow is more serious. The muscle strength of bilateral triceps was Grade V, bilateral biceps brachii muscle strength was Grade V, the bilateral deltoid muscle strength was Grade V, and normal muscular tension of extremities. The bilateral biceps reflex was normal, the bilateral triceps tendon reflex was normal, the bilateral radial artery membrane reflex was normal, and the bilateral knee and Achilles tendon reflex was normal. Hoffman sign in both hands was negative.

**Figure 1:**
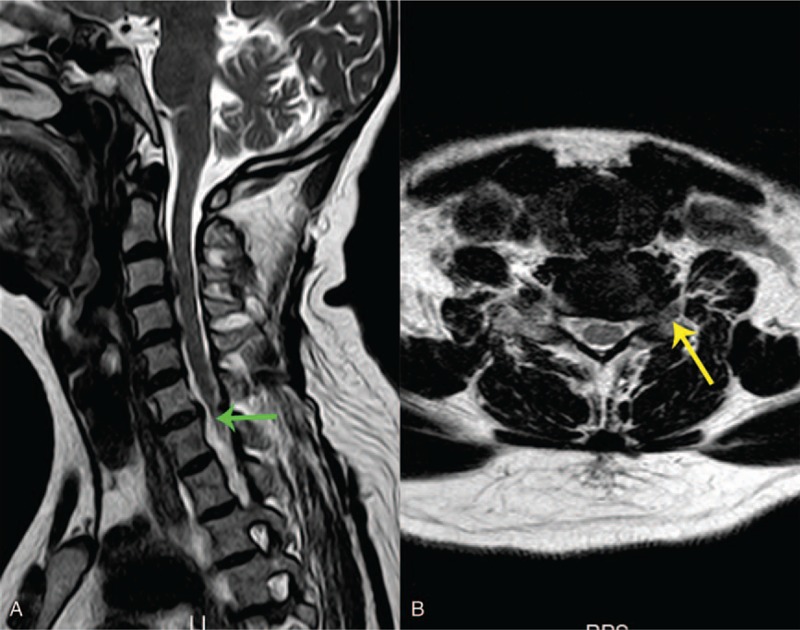
Sagittal (A) and axial (B) of cervical spine magnetic resonance imaging demonstrated that the discs of C3-4 and C6-7 were protruded, and the local dual sac was compressed in an arc. No abnormal signal was found in the spinal cord.

**Figure 2:**
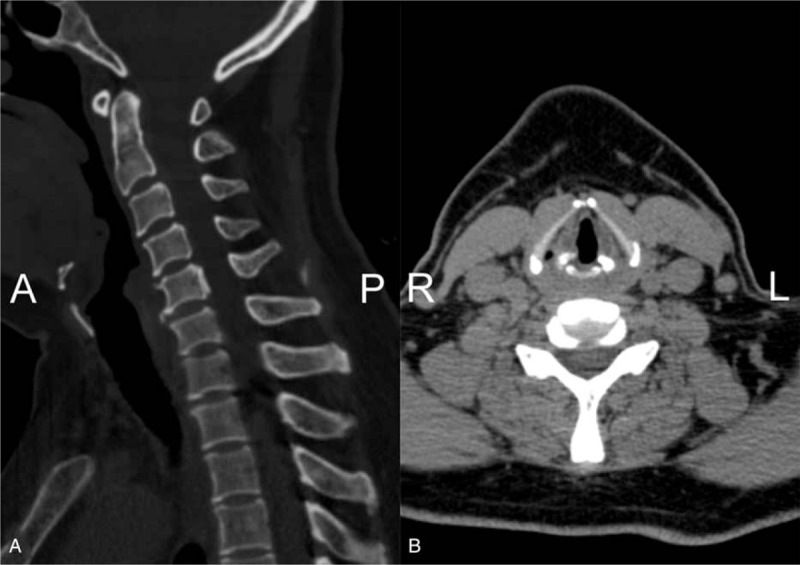
Sagittal (A) and axial (B) of cervical spine CT showed that the cervical curvature became straight.

**Figure 3:**
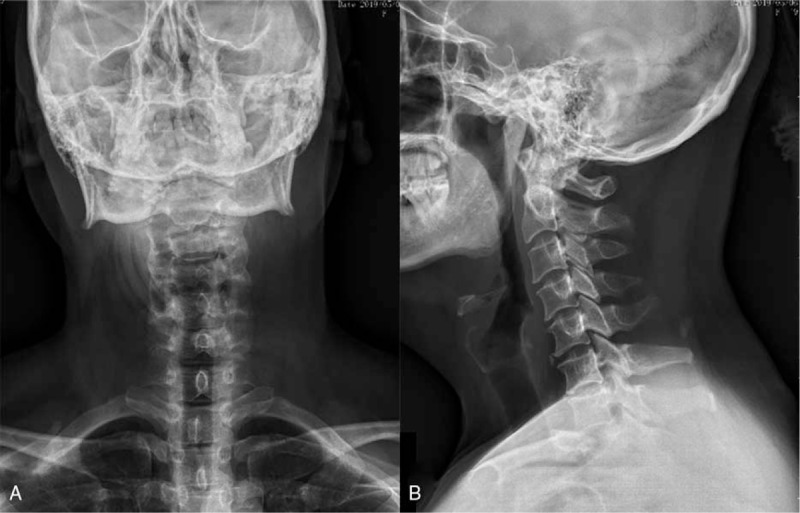
Postero-anterior radiograph (A) and lateral (B) X-ray film of cervical spine showed the cervical curvature became straight.

Magnetic resonance imaging (MRI) showed that C3-4 central disc herniation and C6-7 left far lateral disc herniation (Fig. 1A-B). Three dimensional CT of cervical spine showed that the cervical curvature became straight (Fig. 2A-B) and that the atlantoaxial joint space on both sides was approximately symmetrical. Cervical vertebrae X-ray film displayed that the curvature of cervical spine became straight, the edge of C3-6 was hyperosteogeny, the corresponding intervertebral space was not obviously narrowed, and so was the neck ligament calcification (Fig. 3A-B). Results of electromyography of extremities showed that both motor and sensory nerve conduction velocity of the upper extremities was normal, and the H reflex of the double tibial nerve was normal, however, the biceps brachii, the triceps brachii were displayed as neurogenic damage. The primary diagnosis was CDH (C6-7).

### 3.2 Surgical technique

The patient was placed in prone position. Puncture site was prepared with povidone iodine and covered with sterility, the X-ray examination of the cervical spine was performed to determine the intervertebral space of the C6-7, and 0.2% ropivacaine injection was used for local anesthesia at the puncture area. The position of disposable puncture needle was dynamically observed under the guidance of ultrasound. After the puncture needle reached the corresponding nerve root, the surgeon sucked back the syringe, and no blood or cerebrospinal fluid was found. The surgeon injected 5 ml of ropivacaine, which was 0.2%, and observed whether the patient's discomfort symptoms disappeared.

**Figure 4:**
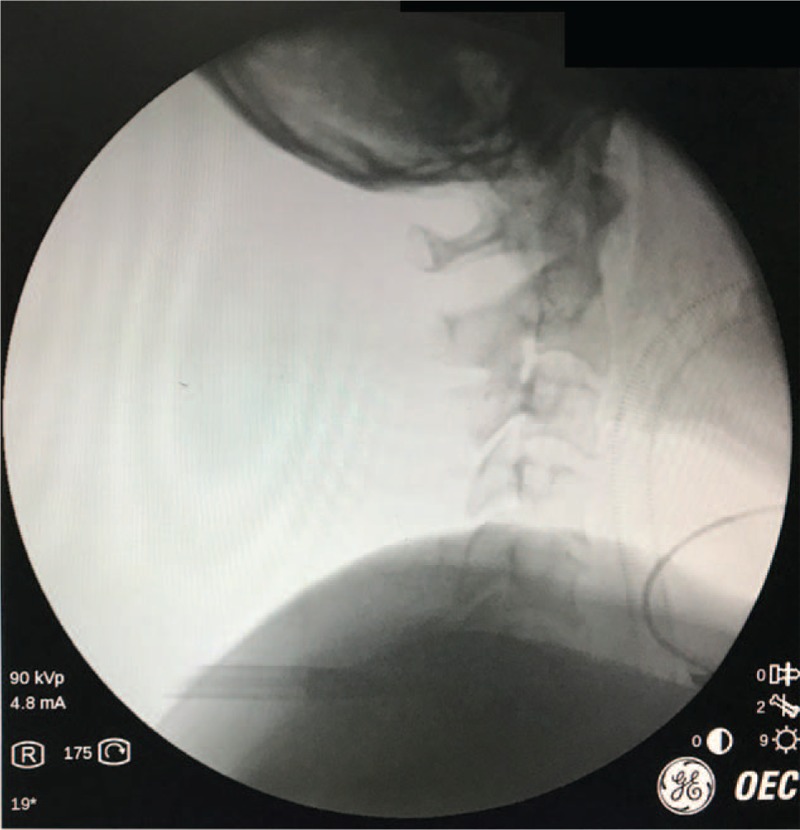
C-arm fluoroscopy was used to install the endoscope sleeve to determine its position in the intervertebral space of C6-7 intraoperatively.

**Figure 5:**
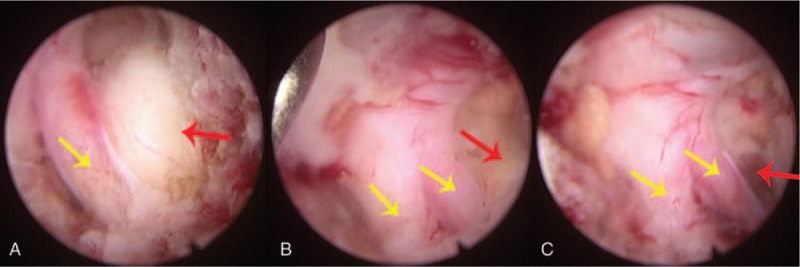
Under endoscopy, (A) with yellow arrows indicating nerve roots and red arrows indicating prominent cervical intervertebral discs, (B) C6-7 double nerve root variation (yellow arrow) on the left side of the spinal cord was observed, (C) the yellow arrow indicates that the C6-7 nerve root has been decompressed, and the red arrow indicates that the prominent cervical intervertebral disc has been removed.

The patient was placed in prone position after general anesthesia. Fluoroscopic C-arm was used to localize the lesion segment (Fig. 4). The surgeon stood on the side of more pressure. Video monitor was placed opposite to the surgeon. Approximately 2 to 3 cm skin incision, about 1 cm lateral to midline was made at the spinal level to be decompressed. The installation of expansion sleeve was carried out step by step. Lamina, facet joint and interlaminar area were identified. Left laminectomy was done using a high-speed drill to thin out the lamina. Good dura pulsation was observed after removal of the ligamentum flavum. Compressed nerve roots, which revealed double nerve root variations in the left side of C6-7 segment, were observed under endoscopy (Fig. 5). We carefully protected the nerve root and removed the prominent nucleus pulposus with nucleus pulposus forceps. After confirming that the nerve root was fully decompressed, the wound was sutured. No antibiotics were used preoperatively or postoperatively. Only under the precondition of cervical brace protection, could the patient get out of bed 6 hours after the surgery, and cervical brace protection should last for 2 weeks. The decompression was evaluated by MRI within 1 week after operation.

**Figure 6:**
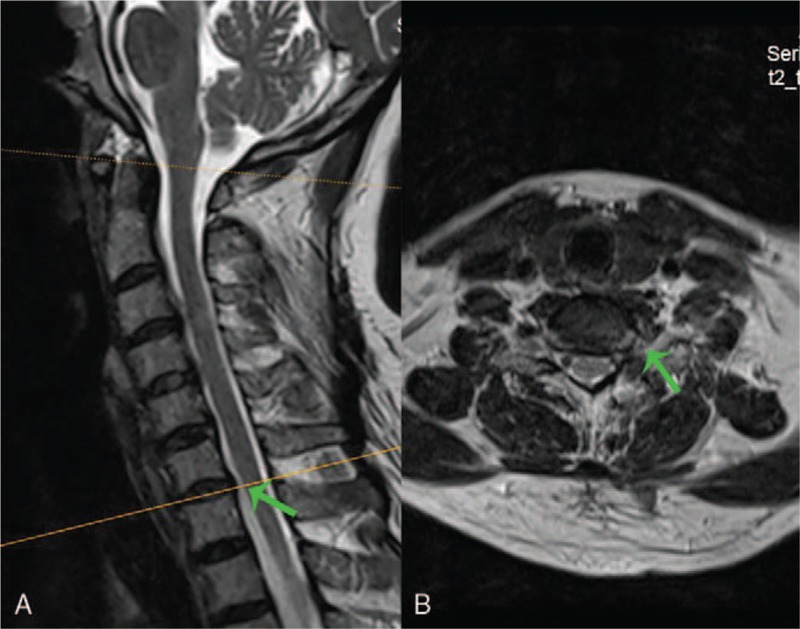
An magnetic resonance imaging examination was performed 1 week after the operation. (A) Sagittal images showed that the dual sac compression at the C6-7 segment was relieved (green arrow), (B) and axial images showed no compression at the left outlet nerve root of C6-7 (green arrow).

The pain symptoms disappeared after the patient was treated with C6-7 nerve root block. Endoscopic displayed C6-7 double nerve root variation on the left side of the spinal cord intraoperative (Fig. 5). An MRI examination was performed one week after the operation. Sagittal images showed that the dual sac compression at the C6-7 segment was relieved (Fig. 6A), and axial images showed no compression at the left outlet nerve root of C6-7 (Fig. 6B). There was no neurological complication postoperatively and no recurrence of CDH during the 5 months’ follow-up period. The hospitalization time was 5 days, the bleeding volume was 52.6 ml and the operation time was 68.2 minutes. At the preoperative and the final follow-up, the physiological curvature of the cervical spine was 8.9° and 8.8°, respectively, the intervertebral height was 1.31 cm and 1.29 cm, respectively, the Visual Analogue Scale score decreased from 6 to 1, the Japanese Orthopaedic Association score improved from 16 to 19, and the sf-36 score increased from 62 to 79 (Table 1).

**Table 1:**
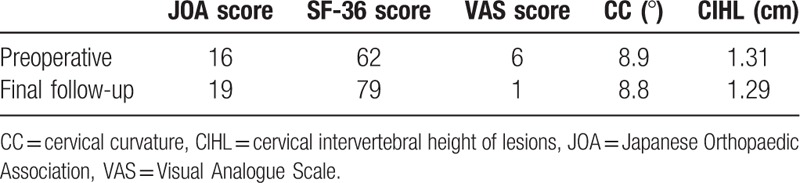
Evaluation parameters were collected preoperatively and postoperatively.

## 4 Discussion

In this case, the symptoms of cervical disc herniation (CDH) are atypical and inconsistent with the results of the imaging examinations, which makes the diagnosis of CDH more difficult. In addition, whether open surgery or minimally invasive surgery should be used in the treatment of cervical spondylosis is still controversial. In this study, we applied the classic function of SNRB to determine the presence or absence of root lesions at the c6-7 segment. After SNRB, we observed the remission or non-remission of radicular pain in the patient's left upper extremity to determine whether the patient is with or without radiculopathy. This patient experienced significant relief of severe pain in the left arm after SNRB, and preoperative electromyography and MRI also indicated C6-7 segment nerve root damage. Finally, with the help of SNRB, we identified the operative segment. Good therapeutic effect has been achieved. The experience is summarized as follows.

Usually, the main symptom of cervical radiculopathy is upper limb radiation pain. The responsible segment is determined by combining medical history, physical examination, signs, and imaging examination. However, the result of image examination was inconsistent with clinical symptom in this study. We applied selective nerve root block to determine the injured of C6 nerve root and pain symptoms disappeared with nerve block. In addition, intraoperative exploration also confirmed C6 nerve root compression. Furthermore, double nerve root variation was found in C6-7 foramen, perhaps this is another reason of stenosis of the nerve root foramen volume. Therefore, we believe that selective nerve block can improve the diagnostic rate of atypical CDH.

Ando was the first to report CSR^[10]^ that can be treated with conservative and surgical treatment. In 1944, Spurling^[11]^ described that intervertebral foramen decompression through posterior approach can be effective and safe for the treatment of CSR, but the complication of cervical pain and muscle sequelae occurred postoperative. In 1958, Smith and Robinson^[12]^ finished the first case of anterior cervical disc resection and fusion surgery, and achieved a good clinical outcome. However, the technique still has some shortcomings, such as recurrent laryngeal nerve paralysis, dysphagia, Horner syndrome, esophageal perforation, cage displacement, cerebrospinal fluid leakage, adjacent segment degeneration, and pseudo-joint formation.^[13–15]^ In 2007, Ruetten et al^[16]^ firstly recommended cervical intervertebral disc excision through posterior approach by percutaneous endoscopic surgery. Endoscopic surgery can achieve the same therapeutic effect as traditional surgery, but shorter hospital stay, less bleeding, and less tissue damage.^[17–19]^ In this study, we attribute the good results to the application of PPECD technology.

The indications and contraindications of PPECD technology are as follows. Indications^[17,20,21]^:

(1) Unilateral CSR, which was ineffective after non-surgical treatment, showed lateral or posterolateral soft disc herniation on imaging, and was consistent with clinical symptoms;
(2) No history of cervical spine surgery;
(3) Those without myelopathy;
(4) Osteogenic stenosis of intervertebral foramen.

Contraindications^[17,20,22]^:

(1) Only neck pain, but imaging showed no obvious CDH or nerve root pressure;
(2) central disc herniation;
(3) cervical disc calcification;
(4) obvious cervical instability, deformity, and cervical back arch;

Endoscopic surgery can be performed under general anesthesia or local anesthesia. General anesthesia was chosen in this case, which enabled the surgery to be completed successfully. General anesthesia can make patients avoid the fear of surgery caused by local anesthesia.

Despite the perfect results achieved in our study, there are also some limitations as follows. First of all, this report is not a large sample study. Therefore, large-scale, multi-center data is needed to evaluate the results. Second, whether multi-level surgery affects biomechanical stability needs further study. At the same time, it ensures the further development and perfection of finite element technology. At last, it is impossible to enlarge the scope of surgery to deal with the lesions in the medial cervical spinal cord or to reconstruct the intervertebral space.

The application of SNRB combined with PPECD for CDH could achieve a satisfactory effect of position and decompression on the injured nerve root. Besides, we recommend that surgery be performed under general anesthesia to minimize patients’ emotional stress and discomfort.

## Acknowledgments

In particular, Tong Yu thanks his wife whose name is Xiwen Zhang for her care, patience, understanding and support, which has given me enough time and passion to do scientific research work.

## Author contributions

**Conceptualization:** Jun Zhang, Hai-Chi Yu, Su-Li Luo.

**Methodology:** Jun Zhang, Hai-Chi Yu.

**Project administration:** Hai-Chi Yu.

**Resources:** Hai-Qing Tian.

**Supervision:** Jiu-Ping Wu, Qinyi Liu.

**Visualization:** Jiu-Ping Wu, Qinyi Liu.

**Writing – original draft:** Tong Yu, Su-Li Luo.

**Writing – review & editing:** Qinyi Liu.
